# Orexin signaling regulates both the hippocampal clock and the circadian oscillation of Alzheimer’s disease-risk genes

**DOI:** 10.1038/srep36035

**Published:** 2016-10-31

**Authors:** Zhixiong Ma, Weiliang Jiang, Eric Erquan Zhang

**Affiliations:** 1 College of Life Sciences, Beijing Normal University, Beijing 100875, China; 2 National Institute of Biological Sciences, Beijing 102206, China; 3 Department of Gastroenterology, Shanghai First People’s Hospital, School of Medicine, Shanghai Jiao Tong University, Shanghai, 200080, China

## Abstract

Alzheimer’s disease (AD) is a circadian clock-related disease. However, it is not very clear whether pre-symptomatic AD leads to circadian disruption or whether malfunction of circadian rhythms exerts influence on development of AD. Here, we report a functional clock that exists in the hippocampus. This oscillator both receives input signals and maintains the cycling of the hippocampal *Per2* gene. One of the potential inputs to the oscillator is orexin signaling, which can shorten the hippocampal clock period and thereby regulate the expression of clock-controlled-genes (CCGs). A 24-h time course qPCR analysis followed by a JTK_CYCLE algorithm analysis indicated that a number of AD-risk genes are potential CCGs in the hippocampus. Specifically, we found that *Bace1* and *Bace2*, which are related to the production of the amyloid-beta peptide, are CCGs. *BACE1* is inhibited by *E4BP4*, a repressor of D-box genes, while *BACE2* is activated by CLOCK:BMAL1. Finally, we observed alterations in the rhythmic expression patterns of *Bace2* and *ApoE* in the hippocampus of aged *APP/PS1dE9* mice. Our results therefore indicate that there is a circadian oscillator in the hippocampus whose oscillation could be regulated by orexins. Hence, orexin signaling regulates both the hippocampal clock and the circadian oscillation of AD-risk genes.

Recent reports have revealed that circadian genes are strongly associated with Alzheimer’s disease (AD)^1^. Researchers have found that circadian rhythms are significantly disturbed in AD and that such disturbance is of significant clinical importance in terms of behavioral symptoms^2^,^3^,^4^,^5^. Molecular clocks located throughout the body in peripheral tissues and cells are organized into a hierarchical system that is ultimately controlled by a master clock located in the suprachiasmatic nucleus (SCN) of the hypothalamus^6^,^7^,^8^,^9^,^10^,^11^. Autonomous circadian rhythms are generated by intracellular transcriptional feedback loops that feature cis-regulatory elements such as E-boxes, D-boxes, and ROR-elements (ROREs). In general, the so-called clock control genes (CCGs) with these cis-regulatory elements in their promoter regions are regulated by transcriptional activators or repressors^7^,^12^,^13^,^14^,^15^.

The deterioration of sleep-wake patterns that results from disturbances in the circadian clock represent some of the most common complaints in elderly human populations, especially in patients with dementia and AD^16^,^17^. AD patients are commonly characterized by the aggregation of the pathogenic amyloid-beta (Aβ) peptide and Tau proteins in the brain^18^,^19^, especially in the hippocampus and cortex regions of the brain. Accumulating evidence has established that the aberrant expression of core clock genes is strongly associated with the pathogenesis of AD^4^,^15^. It is known that the brain-specific knockout of *Bmal1* results in AD-like neurodegeneration in mice^4^,^20^. Polymorphisms in the *CLOCK* gene have been associated with the development of AD in humans^21^,^22^. Rhythmic expression of *BMAL1, CRY1*, and *PER1* are lost in pineal from both preclinical and clinical AD patients^5^. Expression of *Per2* has also been reported to be a blunted diurnal variation pattern in the SCN in old AD mice^23^.

Orexin is a neuropeptide hormone encoded by the *orexin precursor* gene and synthesized in neurons that originate in the lateral hypothalamus (LH). There are two orexin neuropeptides: orexin A and orexin B (OR-A and OR-B). Both of these peptides can bind to two G-protein coupled receptors, *orexin receptor 1* and o*rexin receptor 2*, which are encoded, respectively, by *Hcrtr1* and *Hcrtr2*^24^,^25^. This neuropeptide system plays an important role in numerous behavioral and regulatory functions, including sleep homeostasis and feeding behaviors^26^,^27^. Sleep homeostasis is thought to be crucial to hippocampal-dependent memory formation and consolidation^28^,^29^. Disruption of sleep homeostasis is known to be directly linked to pathological deterioration in AD. A direct piece of evidence linking orexins and AD is the finding that patients with AD showed altered orexin A levels in cerebrospinal fluid (CSF) relative to normal control individuals^2^,^30^. Further, knockout of the *orexin precursor* gene has been shown to reduce the deposition of Aβ in the in hippocampus and cortex of *APP/PS1dE9* mice^3^. Orexin receptors have also been demonstrated to exert a neuroprotective effect in AD via heterodimerization with *GPR103*, another G-protein coupled receptor^24^.

It has been verified that hippocampus-dependent memory impairment is caused mainly by the accumulation of the Aβ peptide and Tau proteins, both in aging and in AD^31^,^32^. The physiological isoform of Aβ originates from the amyloid precursor protein (*APP*) via sequential cleavages that are catalyzed by *BACE1* and *BACE2* and by *Presenilin-1* and *Presenilin-2 (PSEN1* and *PSEN2*). The mechanism of Aβ aggregation has been studied in detail. Recently, the metabolism of Aβ has attracted extensive research attention alongside the re-discovery of the critical function of the *APOE* gene in AD pathology^33^,^34^,^35^,^36^. Aβ levels are known to have a diurnal oscillating pattern that has been found to dynamically correlate with the levels of orexins in CSF^3^,^20^,^37^,^38^. It is also known that the amount of Aβ in CSF increases significantly in the brains of mice during both acute sleep deprivation and following orexin A infusion^38^. It is typically thought that sleep can accelerate the circulation of CSF, leading to a decrease in Aβ levels^37^. However, given that the metabolism of Aβ includes not only its clearance, but also its production and transport, we have for some time suspected that the production and transport of Aβ is related both to circadian rhythms and to orexin signaling.

It remains controversial as to whether or not a clock oscillator exists in the hippocampus^18^,^39^,^40^,^41^,^42^. However, the reported circadian oscillations of the cAMP/MAPK/CREB signaling pathway strongly suggest that there is indeed an oscillator functioning in the hippocampus^43^,^44^,^45^. Other researchers have also reported rhythmicity in the expression patterns of core clock genes in the hippocampus^41^,^46^,^47^,^48^. In this study, using real-time recording of hippocampal slices cultured *ex vivo* combined with pharmacological, genetic, biochemical, and molecular approaches, we confirmed the hypothesis that there is a self-sustained circadian clock in the hippocampus. We also found that the hippocampal clock is a functional clock that can be regulated by inputs such as orexins. Furthermore, we observed that this clock functions to control the transcription of AD-risk genes and that the circadian clock is disturbed by the AD pathology in *APP/PS1dE9* mice. Our results suggest that the pathology of AD is associated with the circadian clock in the hippocampus and further suggest that orexin signaling may have an impact on the production and transport of the AD-related Aβ peptide.

## Materials and Methods

### Animals

All mice used in this paper were housed at 22 ± 2 °C, with 60 ± 5% humidity, and maintained with a LD 12:12 photoperiod (12 h light, 12 h dark, lights on at 07:00). Mice were fed a normal diet and provided water ad libitum. *Clock*^delta19/+^ mice^49^ and homozygous *mPer2*::luciferase knock-in mice (*mPer2*^luc/luc^)^50^ were purchased from the Jackson Laboratory. *Clock*^delta19/+^ mice were crossed to *mPer2*^luc/luc^ reporter mice. From heterozygous offspring, we created double homozygous *Clock*^delta19/delta19^; *mPer2*^luc/luc^ mice. In this study, *APP/PS1dE9* transgenic mice were used to evaluate the mechanism through which the circadian clock contributes to AD^51^. These mice express a chimeric mouse/human *APP (Mo/HuAPP695swe*) and a mutant human *PSEN1 (PS1-dE9*). *APP/PS1dE9* mice were also crossed with *mPer2*^luc/luc^ reporter mice to create *APP/PS1dE9*; *mPer2*^luc/luc^ mice. *mPer2*^luc/luc^, *Clock*^delta19/delta19^; *mPer2*^luc/luc,^ and *APP/PS1dE9*; *mPer2*^luc/luc^ mice were generated for hippocampal dissection and real-time recoding of the hippocampal oscillation. All experiments for this study were carried out with 2–4 month old male mice, except as otherwise noted. Wild-type (WT) mice were maintained in a LD 12:12 photoperiod condition with free access to food and water for 2 weeks before being kept in complete darkness (DD) for an additional 48 h. WT Mice (n = 3) were sacrificed every 4 h throughout the course of one circadian cycle (both under LD and DD condition). The hippocampus were dissected quickly from brains. Hypothalamus samples were collected every 6 h for one circadian cycle from young (age 4 months, n = 3–5) or aged (aged 12–15 months, n = 3–5) WT and *APP/PS1dE9* transgenic mice brains. Animal experiments were performed in accordance with the NIBS institutional regulations, after approval by the Institutional Animal Care and Use Committee (IACUC).

### Preparation of hippocampus slices

*mPer2*^luc/luc^, *Clock*^delta19/delta19^; *mPer2*^*l*uc/luc^, and *APP/PS1dE9*; *mPer2*^luc/luc^ mice were anesthetized with 2,2,2-Tribromoethanol (Sigma) and sacrificed at ZT12-15 to reveal the bioluminescence rhythm in the hippocampus; these protocols were performed as previously described^49^,^50^. The brain was rapidly removed from the mouse and placed in ice-cold Hanks’ balanced salt solution (HBSS) (Thermo Fisher, pH = 7.2–7.4). The brain was then cut into slices of 220 μm thickness with a vibrating-blade microtome (VT1000S, Leica Microsystems). The slices were maintained in ice-cold HBSS during this procedure until the point when explants were placed into the experimental medium for luciferase recording. The hippocampus was carefully and quickly isolated from the brain slices using scalpels and was then explanted onto a culture membrane (Milli-CM 0.4 μm, EMD Millipore) on top of the liquid surface of a 35 mm Petri dish (Corning) and sealed with a greased 40 mm coverslip. Samples were then cultured with 1.3 mL of HEPES-buffered explant medium supplemented with 1 μM luciferin (Promega) and B-27 supplements (Thermo Fisher). The explants were incubated at 36 °C, and bioluminescence was monitored for one minute in each 10-minute interval using a dish-type luminometer (Actimetrics). The assessment of circadian periods and phases were performed as described in previous reports^49^,^50^,^51^.

### Cell culture and transfection

HEK293 cells were grown in regular DMEM supplemented with 10% FBS (Hyclone, GE Healthcare Life Sciences) and antibiotics at 37 °C, 5% CO_2_. For transfection, rapidly growing cells were trypsinized and re-suspended in DMEM containing 10% FBS lacking antibiotics at a 0.1 × 10^6^ cells/ml concentration. We next added 50 μl of transfection reagent mixture (0.5 μl/well Lipofectamine 2000 in Opti-MEM; Thermo Fisher) to wells containing pre-spotted plasmids. We incubated the wells at room temperature for 20 min and subsequently added 100 μl of cells (0.1 × 10^5^ cells/well). Approximately 6 h after transfection, we replaced this medium with 150 μl of pre-warmed fresh DMEM containing 10% FBS and antibiotics and allowed the cells to grow for an additional 24–30 h. 36 h post-transfection, we replaced this medium with 150 μl of HEPES-buffered explant medium supplemented with luciferin (1 μM) and B-27 supplements; the plates were sealed with an optically clear film. We next loaded these plates into a 36 °C incubator and recorded bioluminescence expression with an Infinite^®^ 200 PRO series microplate reader (Tecan, Thermo Fisher).

### Plasmid DNA and materials

The hippocampal slices were treated with final concentrations of 10, 50, and 100 nM orexin A (Abcam) dissolved in DMSO. Forskolin (Sigma) was dissolved in DMSO and the hippocampal slices were treated with a final concentration of 10 μM. Orexin B (Genscript) was dissolved in DMSO; the hippocampal slices were treated with a final concentration of 500 nM. EMPA (Sigma), a high-affinity, reversible, and selective *Hcrtr2* antagonist, was dissolved in DMSO^52^; the hippocampal slices were treated with a final concentration of 10 μM. All compounds, drugs and peptides were titrated in the explant medium to the final concentration and then the prepared medium was added to the 35-mm Petri dish with the slices on the insert. To express *E4BP4*, the coding sequence of the *E4BP4* gene (NM_001289999.1) was amplified from cDNA and subcloned into the pcDNA3.1 plasmid (Thermo Fisher). The human 1.0-kb *BACE1*-promoter (NC_000011.10) and the 1.4-kb *BACE2*-promoter (NC_000021.9) were amplified from DNA extracted from HEK293 cells; these amplification products were cloned as pGL3-basic plasmid reporter constructs (Promega) and named, respectively, P(*BACE1*)*-luc* and P(*BACE2*)*-luc*. The primers used for the PCR amplification of target sequences are detailed in Supplementary Table 4.

### RNA isolation and quantitative real-time PCR

Total RNA was extracted from the hippocampus and hypothalamus using Trizol reagent (Thermo Fisher). A 500 ng aliquot of total RNA was reverse transcribed into cDNA using PrimeScript™ RT Master Mix (Takara) and then analyzed with SYBR GREEN qPCR mix (Kapa Biosystems) using a CFX96 instrument (Bio-Rad). The relative quantification of expression levels was performed using a previously-described ^ΔΔ^CT calculation method^53^. *Beta-Actin* was used as a reference gene. The specific primer pairs used for the analysis of the core clock genes and the AD-risk genes were designed using Primer3 (Supplementary Tables 1–3).

### Statistical analysis

OriginPro 2016 software (OriginLab) was used for statistical analyses. Period change data were analyzed with one-way/two-way ANOVA followed by Tukey’s HSD test. To validate whether or not the AD-risk genes displayed circadian oscillations under the LD and DD conditions in the hippocampus, we measured 24 h oscillations in transcript abundance using the JTK_CYCLE algorithm; we set a 5% false discovery rate for detection^1^,^54^. We have here reported the results as means with the standard error of the mean (mean ± s.e.m.), and have used *P* < 0.05 as the criterion for evaluating the inferential statistical significance of differences.

### Immunofluorescence

Mice were anesthetized with 2,2,2-Tribromoethanol and fixed by perfusion of 4% paraformaldehyde. Whole brains were dissected and post-fixated with 4% paraformaldehyde for an additional 2 h before dehydration in a 30% sucrose solution overnight at room temperature. Thoroughly dehydrated whole brains were frozen in powdered dry ice. Brain tissues were sliced using a cryostat instrument (CM3050S, Leica) to a thickness of 45 μm. The sections were stained with anti-orexin A (1:125, Santa Cruz) overnight at 4 °C, then incubated with Alexa Fluor 594 labeled anti-goat IgG (1:500, Thermo Fisher) and DAPI (Sigma, 1 mg/mL, 1:1000) for 1 h at room temperature in darkness. Images were obtained via confocal microscopy (Zeiss confocal LSM800). To visualize fibrillary amyloid plaques, sections were stained with Thioflavin T^55^,^56^. Images were obtained with a virtual scanning system for microscopy slides (Olympus VS120).

## Results

### A self-sustained circadian oscillator exists in the hippocampus

**Figure 1:**
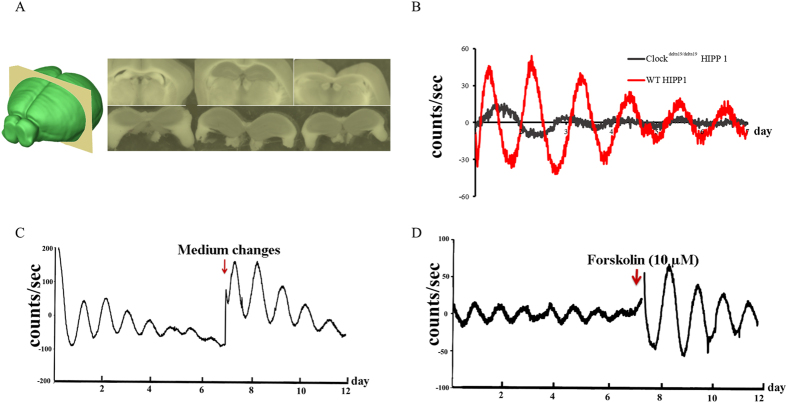
A self-sustained circadian oscillator exists in the hippocampus. (**A**) Dissected hippocampus slices in ice-cold 1 × HBSS buffer (PH = 7.2–7.4). (**B**) Deficiency of *Clock* leads to the attenuation of circadian rhythms in the hippocampus. We recorded the real-time luciferase activity of *Clock*^delta19/delta19^; *mPer2*^luc/luc^ and WT control hippocampus slices and found an attenuation of circadian rhythms in *Clock* mutants. (**C**) The oscillation of the hippocampus slices was damped after one week in *ex vivo* culture conditions. However, medium changes at days 7–8 induced synchronization of hippocampal oscillation (**D**) Forskolin (10 μM) treatment induced synchronization of hippocampal oscillation.

We first evaluated whether there was a self-sustained circadian oscillator in the hippocampus. The hippocampus of *mPer2*^luc/luc^ mice, which we were able to isolate completely from other brain regions (Fig. 1A), clearly maintains oscillations in *ex vivo* culture (Fig. 1B–D). We found that the oscillations could be synchronized by changing the growth medium and by treatment with forskolin, which activates adenylyl cyclase and then triggers cyclic AMP signaling (Fig. 1C,D). After one week in *ex vivo* culture, the hippocampal oscillation is dampened and becomes de-synchronized (Fig. 1C,D). This oscillation could be synchronized by treatment with 10 μm forskolin. We also found that *Clock* malfunction results in defects in maintaining the hippocampal oscillator. *Clock*^delta19/delta19^; *mPer2*^luc/luc^ mice carry a mutation in the *Clock* gene that results in a dominant-negative protein that cannot activate transcription^57^. We dissected the hippocampus and recorded the oscillation of the *Clock*^delta19/delta19^; *mPer2*^luc/luc^ mice. We found that deficiency in *Clock* led to an attenuation of circadian rhythms in the hippocampus (Fig. 1B).

**Figure 2:**
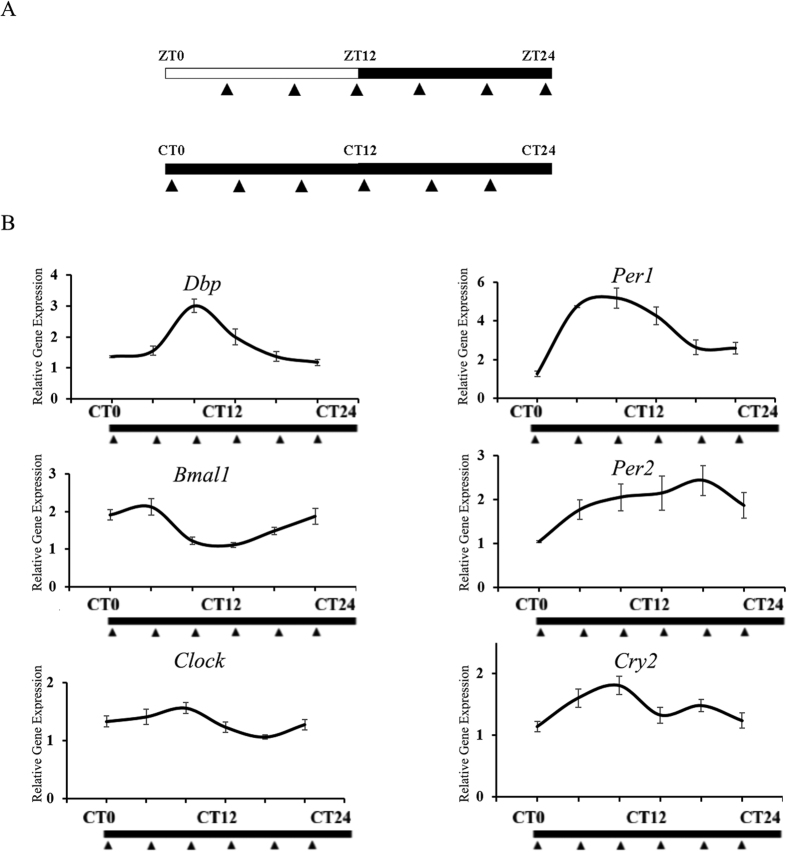
qPCR and JTK_CYCLE algorithm analysis of core clock gene in hippocampus. The qPCR results and the cycling parameters predicted by the JTK_CYCLE algorithm of core clock genes under the DD condition in the hippocampus. (**A**) Schedule for sampling in the LD and DD conditions. WT mice were decapitated at ZT4, 8, 12, 16, 20, and 24 under the LD condition or at CT0, 4, 8, 12, 16, and 20 under the DD condition; (**B**) Gene expression patterns of core clock genes in the hippocampus in the DD condition (*Dbp, Bmal1, Per1, Per2, Clock*, and *Cry2*). The qPCR primers used here are listed in Supplementary Tables 2 and 3.

To confirm our finding that a self-sustained circadian oscillator exists in the hippocampus, qPCR analysis was conducted to measure the expression levels of core clock genes in the hippocampus in the DD condition. We used the JTK_CYCLE algorithm to characterize cycling variables, including period, phase, and amplitude, for the core clock genes. The expression of 6 out of 7 core clock genes tested here (with the exception being *Cry1*) exhibited circadian rhythms (Fig. 2B and Table 1), indicating that an intact oscillator exists in the hippocampus. The JTK_CYCLE algorithm results revealed an exact 24 h period for the expression of core clock genes in the hippocampus (Table 1). The mRNA acrophase of *Clock* and *Bmal1,* as predicted by the JTK_CYCLE algorithm, occurred at CT0 and CT6, respectively; the JTK_CYCLE algorithm-predicted mRNA acrophase of *Per1, Per2, Dbp,* and *Cry2* occurred, respectively, at CT10, CT14, CT0, and CT10 (Table 1).

### Treatment with orexins shortens the period of the hippocampal oscillator

**Figure 3:**
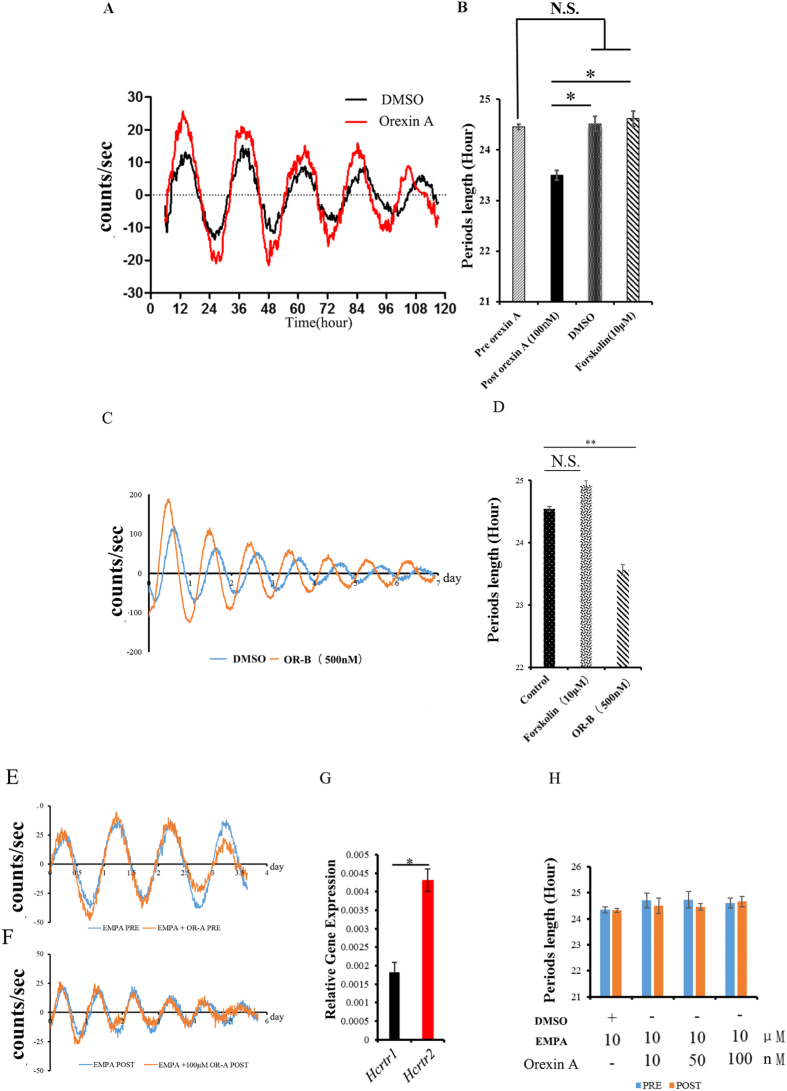
Orexin treatment led to shortening of the period of the hippocampal oscillator. (**A**) Effects of orexin A and DMSO treatments on hippocampal oscillation. (**B**) Orexin A treatment induced shortening of the period of the hippocampal oscillator as compared with the no-treatment control, the DMSO control, and the Forskolin (10 μM) treatment (n = 4–9); Bar graph (mean ± s.e.m.) of period; length of periods was analyzed by two-factor ANOVA, followed by Tukey’s HSD *P < 0.05. (**C**) Effects of orexin B and DMSO treatments on hippocampal oscillation. (**D**) Orexin B treatment induced shortening of the period of the hippocampal oscillator as compared with the DMSO control and the Forskolin (10 μM) treatment (n = 4–18); Bar graph (mean ± s.e.m.) of period; length of periods was analyzed by one-way ANOVA, followed by Tukey’s HSD *P < 0.05. (**E,F,H,G**) EMPA blocks the orexin A-induced shortening of the period of the hippocampal oscillator. The period showed no difference before EPMA and orexin A treatment. (**E**) EMPA blocks the orexin A-induced shortening of the period of the hippocampal oscillator. (**F**,**G**) Expression analysis of the orexin receptors shows that *Hcrtr2* is the major receptor in the hippocampus.

Intact circadian oscillators can be regulated by input signals such as hormones, metabolites, and neuropeptides^7^. We evaluated whether oscillation in the hippocampus can be regulated by such signals. Orexins are essential regulators of sleep/wake cycles and are typically thought to be involved in the circadian clock, hippocampus-dependent social memory, and Aβ pathology. We therefore hypothesized that orexin signaling might be able to regulate the hippocampal oscillator. As expected, we found that treatment with orexin A or B (OR-A and OR-B, for short) did indeed regulate the hippocampal clock, leading to a shortened period of hippocampal oscillation in *ex vivo* culture (Fig. 3A). The period of explants was 23.68 ± 0.12 h with 100 nM OR-A treatment and was 24.4 ± 0.09 h with control DMSO treatment (Fig. 3B, Supplemental Fig. 1A–C). Treatment with 500 nM OR-B also shortened the period of hippocampal oscillation *ex vivo* (Fig. 3C). The period of explants was 23.57 ± 0.08 h with 500 nM orexin B treatment and was 24.54 ± 0.03 h with control DMSO treatment. Treatment with 10 μM forskolin did not alter the period of the hippocampal oscillator (Fig. 3C,D).

qPCR analyses of orexin receptor genes revealed that *Hcrtr2* levels are higher than *Hcrtr1* levels in the hippocampus, suggesting that *Hcrtr2* is likely the primary receptor for orexins in this brain region (Fig. 3G). To block the binding of orexins to the receptors, we performed co-treatment experiments with hippocampus slices that tested combinations of EMPA and OR-A or OR-B. In the presence of EMPA, both OR-A and OR-B completely failed to shorten the period of the hippocampal clock (Fig. 3E,F,H; Supplemental Fig. 1D,E). All of these results demonstrate that both the OR-A peptide and the OR-B peptide can function, in redundant roles, in shortening the hippocampal clock.

### Circadian oscillation of Alzheimer’s disease-risk genes

**Figure 4:**
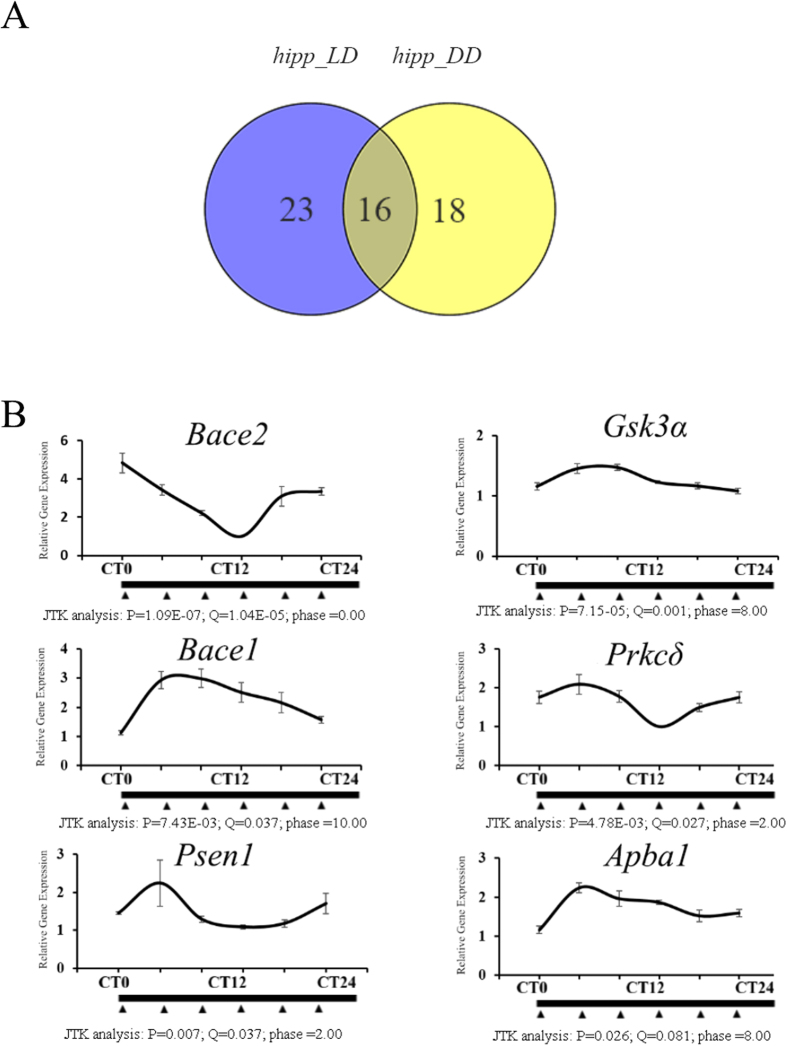
qPCR results and Venn diagram of commonly rhythmic expressed genes in hippocampus under different light conditions. (**A**) Genes that are predicted by the JTK_CYCLE algorithm to be potentially rhythmically expressed under both the LD condition and the DD condition in the hippocampus. (**B**) The expression patterns of *Bace1, Bace2, Psen1, Prkcδ, Gsk3α,* and *Apba1* in the hippocampus in the LD condition; the cycling parameters predicted by the JTK_CYCLE algorithm are listed under the graph of the relative expression of each gene. The qPCR primers used for the expression analysis of the target genes are detailed in Supplementary Tables 1 and 3.

The key AD-risk gene *Psen1* has previously been shown to have rhythmic expression in the liver, which is thought to possess a functional circadian oscillator^1^. Since AD occurs in the hippocampus and the cerebral cortex of the brain, not in peripheral organs, we sought to measure whether, or how, the clock regulates the expression of AD-risk genes in the hippocampus. We therefore monitored the expression of a group of 77 candidate genes (AD RT2 Profiler PCR Array, QIAGEN)^58^,^59^ that are known to be involved in most of the cellular AD-related signaling pathways (Supplementary Table 1) to determine whether these genes merit classification as CCGs. The JTK_CYCLE algorithm was used to identify and characterize the cycling variables of the qPCR dataset for these 77 candidate genes. We found that nearly half of the AD-risk genes exhibited rhythmic expression patterns. We found that 39 and 34 of the candidate genes had rhythmic expression patterns in the LD condition and the DD condition, respectively (Fig. 4A). To narrow down the size of our target gene list, we concatenated the genes that were classified by JTK_CYCLE to be CCGs under both LD and DD conditions. We found that at least 12 out of 77 AD-risk genes are under circadian control in the hippocampus (Fig. 4A; Table 2). *Gnb5, Sncβ, Casp3, ApoE, Cdc2, Bace1, Gng1, Psen2, Gsk3α, Apba1, Bace2*, and *Prkcδ* are the genes most likely to be under clock regulation, as the expression of all of these genes was found to be rhythmic under both the LD and DD conditions in the hippocampus, and the statistical confidence levels for all of their predicted JTK_CYCLE algorithm cycling variables were high (Fig. 4B; Table 2).

### Bace1 and Bace2 are directly regulated by the hippocampal clock

**Figure 5:**
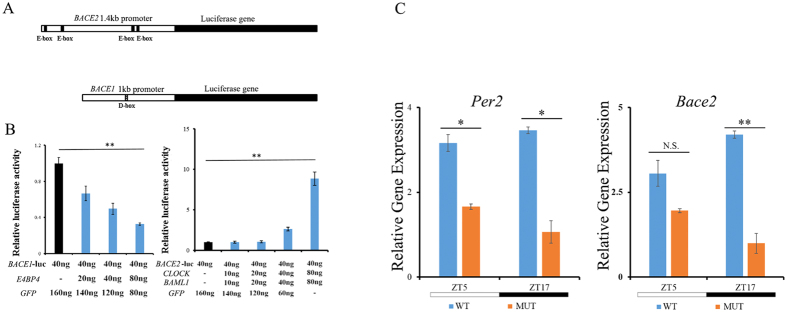
Expression of the *BACE* genes is controlled by the clock. (**A**) Schematic plot of the promoters of the *BACE* genes, including four possible E-boxes located in the 1.4-kb *BACE2* promoter and one D-box located in the 1-kb *BACE1* promoter. (**B**) Left pane: transfection of *E4BP4* inhibited the transcription of *Bace1* in a dose-dependent manner. Right pane: The *CLOCK:BMAL1* complex activated the expression of *Bace2* in a dose-dependent manner. (**C**) The expression of *Per2* and *Bace2* between *Clock* mutant and WT mice in hippocampus. *Clock*^delta19/delta19^ and WT mice (n = 3) were sacrificed at ZT5 and ZT7 under the LD condition; the expressions of *Per2* and *Bace2* were decreased at ZT5 and ZT17 in the *Clock*^delta19/delta19^ mice due to the deficiency of the *Clock* gene; Bar graph (mean ± s.e.m.) of relative gene expression (n = 3, N.S. *P* > 0.05; *P < 0.05; **P < 0.01, student’s t-test).

The expression of both *Bace1* and *Bace2* was classified as rhythmic in the hippocampus based on our qPCR analysis. We found that the promoter regions of *BACE1* and *BACE2* contain putative cis-elements that are potential binding sites for core clock genes. There is a putative D-box in the promoter region of *BACE1* (Fig. 5A). There are four potential E-boxes in the promoter region of *BACE2* (Fig. 5A). We sought to confirm, *in vitro,* whether the *BACE1* and *BACE2* promoters could be regulated by core clock transcription activators such as *CLOCK:BMAL1* and ROREs or be regulated by a repressor like *E4BP4*. Our co-transfection experiments showed that *P*(*BACE2*)*-Luc* expression was activated by the *CLOCK:BMAL1*, and we found that the activation of *BACE2* depended strictly on the amount of *CLOCK:BMAL1* present (Fig. 5B, right panel). *P*(*BACE1*)*-Luc* expression was inhibited by the *E4BP4* repressor (Fig. 5B, left panel). Further qPCR analysis found that *Bace2* decreased the expression at ZT5 and ZT17 in *Clock* mutant mice, as did the canonical E-box gene (Fig. 5C). All these data confirm that *Bace1* and *Bace2* are potential CCGs and could be directly regulated by the hippocampal clock.

### The regulation of E-box genes is crucially important in Alzheimer’s disease

**Figure 6:**
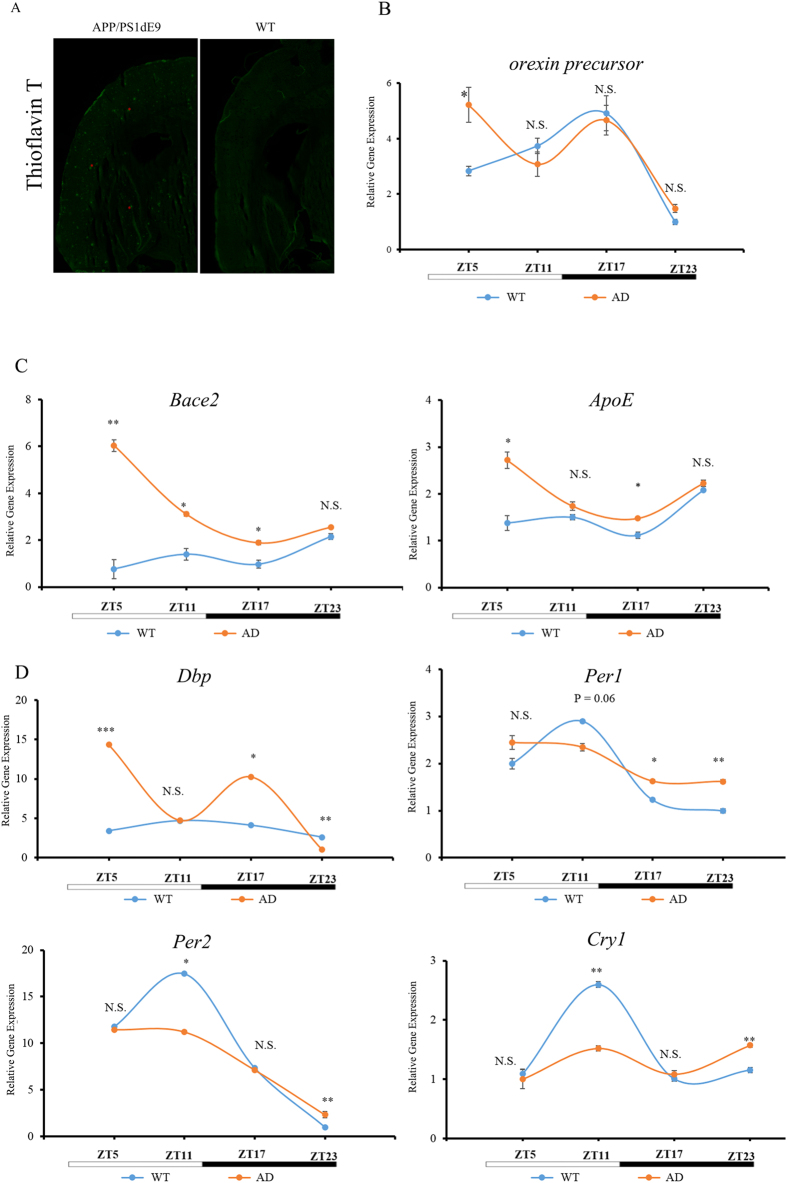
The rhythmicity of *Bace2* and *ApoE* expression is altered in the hippocampus of *APP/PS1dE9* mice. (**A**) Thioflavin T staining of WT and *APP/PS1dE9* mice. Fibrillary amyloid plaques existed only in the brains of the *APP/PS1dE9* mice (Red asterisks indicate fibrillary amyloid plaques). (**B**) The diurnal expression pattern of the *orexin precursor* gene is altered in the hypothalamus of *APP/PS1dE9* mice. Expression of *orexin precursor* mRNA was analyzed by qPCR in the hypothalamus of WT and *APP/PS1dE9* mice. The expression of this gene was higher at ZT5 in *APP/PS1dE9* mice compared with aging WT mice; we did not detect any differences in mRNA levels at other time points. (**C**) The rhythmicity of *Bace2* and *ApoE* expression is altered in the hippocampus of *APP/PS1dE9* mice. Left panel: expression of *Bace2* shows that this AD-related gene increased dramatically in the hippocampus of *APP/PS1dE9* mice. Right panel: *ApoE* gene expression elevated the expression at ZT5, ZT11, and ZT17. The expression of this gene, which is highly correlated with the metabolism of Aβ, increased slightly in the hippocampus of *APP/PS1dE9* mice. (**D**) Canonical E-box genes such as *Dbp, Per1, Per2*, and *Cry1* changed the rhythmicity in the hippocampus of *APP/PS1dE9* mice (n = 3–5, N.S. *P* > 0.05; *P < 0.05; **P < 0.01, student’s t-test). Top left panel: *Dbp*; Top right panel: *Per1*; bottom left panel: *Per2*; bottom right panel: *Cry1*.

The deposition of the Aβ peptide is a major pathological aspect of AD. We confirmed this phenotype in our AD-model mice (*APP/PS1dE9*). Thioflavin T (TFT) is a benzothiazole dye that exhibits enhanced fluorescence upon binding to amyloid fibrils^60^. Our TFT staining results indicated that there were massive amyloid fibrils in the cortex and hippocampus regions of *APP/PS1dE9* mice compared with WT controls (Fig. 6A). We next used qPCR analysis to examine the expression patterns of core clock genes and AD-risk genes, and evaluated how AD progression affected the expression profiles of these genes. As predicted, aged AD mice experience changes in the expression of risk genes, including *Bace2* and *ApoE*. Interestingly, a subset of the tested core clock genes of aged *APP/PS1dE9* mice, including *Per1, Per2, Cry1,* and *Dbp,* had altered expression patterns compared with WT controls. *Dbp* expression was elevated at ZT5 and ZT17 in the hippocampus of aged *APP/PS1dE9* mice, but was decreased at ZT23 compared with WT control; however, the expression of *Per1, Per2, and Cry1* was decreased at ZT11 but elevated at ZT23 (Fig. 6D). Furthermore, the expression of *Bace2* and *ApoE,* both of which have been verified by us and by other researchers to be E-box genes that contain non-canonical E-box motifs in their promoter regions^61^, was also altered in the hippocampus of aged *APP/PS1dE9* mice at ZT5, ZT11, and ZT17 (Fig. 6C). Non-E-box-containing core clock genes, including *Bmal1* and *Clock,* showed only minor, if any, changes in their mRNA levels (Supplemental Fig. 3A,C). There were no differences between aged *APP/PS1dE9* mice compared with WT controls in the expression of *Nr1d1* and *Nr1d2* (Supplemental Fig. 3B), two additional E-box genes, possibly because these genes, which are representative of a secondary loop in the circadian network^62^, are not susceptible to the AD pathology.

### Elevated orexin levels in the hypothalamus of Alzheimer’s disease mice

Finally, we wondered whether the expression of orexins in the brain is governed by the clock. Intriguingly, the expression of the *orexin precursor* gene was found to be rhythmic in the hypothalamus (Fig. 6B and Supplemental Fig. 2A). Further immunofluorescence experiments confirmed that orexin A exhibits the same diurnal expression pattern at the protein level: In the lateral hypothalamus area, the immunostaining signal for the orexin A peptide is higher at ZT17 (Supplemental Fig. 2B), a finding consistent with our qPCR analysis of the *orexin precursor* gene (Supplemental Fig. 2A). Interestingly, the expression pattern of the *orexin precursor* gene is altered in the hypothalamus in *APP/PS1dE9* mice. The transcription of this gene is higher at ZT5 in *APP/PS1dE9* mice than in WT control mice (Fig. 6B). At other time points, the expression of this gene did not differ significantly between the two mouse genotypes (Fig. 6B).

## Discussion

It is well established that the central clock oscillator is located in the SCN. This clock oscillator can run independently and robustly in *ex vivo* conditions for months^50^. However, the hypothesis of a single central clock has been challenged by the discovery of self-sustained oscillators in several tissues, including the liver, lung, and kidney. Even in the mammalian brain, it has been reported that circadian oscillation exists in the amygdala, arcuate nucleus, bed nucleus of the stria terminalis, dorsomedial hypothalamus, habenula, lateral hypothalamus, olfactory bulb, pineal gland, and pituitary gland, as well as SCN^14^. It seems plausible that these peripheral oscillators may be particularly relevant for localized rhythmic events. Here, we confirmed that there is an intact and functional oscillator in the hippocampus. We also observed that *Clock* deficiency impairs local oscillation in hippocampus. Our real-time recording results for the hippocampal oscillation of *Clock*^delta19/delta19^; *mPer2*^luc/luc^ mice were in line with reports claiming that the genetic disruption of the *Clock* impaired local oscillation in the SCN^10^,^57^.

An intact and functional oscillator is typically held to include three major components: a transcription and translation feedback loop (TTFL) oscillator, input pathways, and output pathways. Considering that AD patients are known to have sleep/wake abnormalities related to malfunctions in their orexin systems^2^,^3^,^30^,^38^,^63^, we tested the effect of orexins on the hippocampal oscillator and evaluated whether orexin signals function as inputs for the clock. We found that orexins are indeed involved in regulating the hippocampal oscillator. Surprisingly, these inputs can speed the hippocampal oscillator, resulting in a shortened period. Consistently, real-time recording of the hippocampal oscillator in the aged *APP/PS1dE9*; *mPer2*^luc/luc^ mice revealed that there is indeed a shortened period in these mice (Supplemental Fig. 1F,G). qPCR analysis of the *orexin precursor* gene also indicated that there were higher orexin levels in the hypothalamus area of aged *APP/PS1dE9* mice than in WT control mice (Fig. 6B). All of these results show that a shorter period is a potential intrinsic alteration of the hippocampal circadian oscillator that accompanies the AD pathology.

Phosphorylation of the PERs is known to contribute in the determination of the period length of the circadian clock^64^,^65^. Many pathways regulate circadian timing by altering the phosphorylation status of the PERs. For example, phosphorylation at Ser47 in *Drosophila PER* and dephosphorylation at Ser662 in human *PER2* have been verified as period-shortening molecular events^66^,^67^. Orexins can bind to selective G-protein-coupled receptors and activate kinases and phosphatases^24^,^27^, which may then be involved in regulating the phosphorylation and dephosphorylation of critical amino acid residues in *Per2* that result in a shortening of the period of the hippocampal oscillator^64^,^65^. Previous studies have shown that a deficiency in the levels of orexins in *APP/PS1dE9* mice led to a reduction in the amount of fibrillary amyloid plaque in the cortex and hippocampus as compared to control mice^3^. We also confirmed that there were modest changes, such as period shortening, of the hippocampal clock in aged *APP/PS1dE9* mice (Supplemental Fig. 1F,G), which might have been caused by chronic elevated orexin levels in these mice. Our observations that a higher expression level of the *orexin precursor* gene accompanies the deterioration of amyloid deposition in *APP/PS1dE9* mice, and that high-orexin treatment can shorten the oscillation period of the hippocampal circadian clock, are consistent with the results of these previous studies.

Negative transcription feedback loops are a core mechanism underlying the circadian clock. We questioned whether the transcription of AD-risk genes could be understood as outputs of the hippocampal oscillator. Using the JTK_CYCLE algorithm, a number of AD-risk genes were identified and found to have rhythmic expression profiles. There are two major pathways known to be involved in AD pathology: One includes the genes related to Aβ metabolism (its generation, oligomerization, clearance, and degradation); the other is the pathway for the hyperphosphorylation of the Tau protein^33^,^68^. In our study, genes from both of these pathways were observed to have rhythmic expression patterns. Such genes related to Aβ generation, oligomerization, clearance, and degradation include *Bace1, Bace2*, and *ApoE*. Such genes related to hyperphosphorylation of the Tau protein include *Gsk3α* and *Prkcδ*.

It is known that *BACE1* and *BACE2* are required for the production of the Aβ peptide; these genes are considered to be central to the pathogenesis of AD. *APOE* was previously thought to be a cholesterol transporter^69^,^70^. Intriguingly, *ApoE,* which was recently verified as a regulator of Aβ metabolism^33^,^34^,^71^, has rhythmic expression. The rhythmicity of *Bace1, Bace2*, and *ApoE* expression has been proposed to contribute to the diurnal pattern of Aβ to at least some extent^38^,^72^,^73^,^74^. These suppositions are supported by reports that the acrophase of *Bace2* coincides with the phase of Aβ in CSF^38^,^75^. Although it remains controversial as to whether decreased or increased orexin levels lead to deterioration in AD^2^,^3^,^30^,^38^, many recent studies have assumed the increasingly common view that, at a minimum, orexin levels are disturbed in AD. Our findings indicate that orexin expression exhibits a diurnal pattern, and our data showing elevated expression levels of the *orexin precursor* gene at ZT5 in the hypothalamus area of aged *APP/PS1dE9* mice have led us to prefer the view that higher orexins levels lead to deterioration in AD. Our observation of higher orexin levels in the hypothalamus area is consistent with previous results showing that A) knockout of the *orexin precursor* gene in *APP/PS1dE9* mice led to decreased amounts of amyloid fibrils^3^, and B) that higher orexin levels are correlated with higher Aβ levels^37^,^76^.

**Figure 7:**
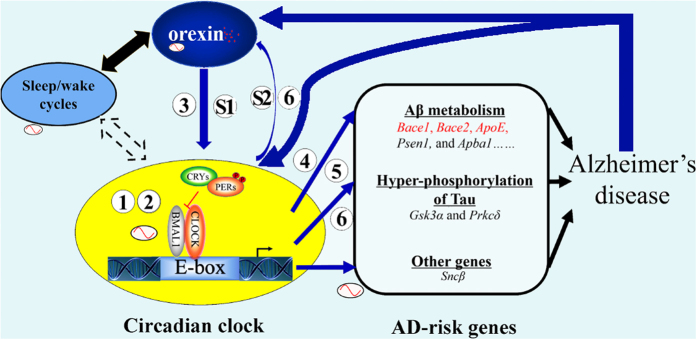
A hypothetical model for the reciprocal control of the hippocampal oscillator. Orexin signaling and AD-risk genes. (1) and (3) A self-sustained circadian oscillator exists in the hippocampus; (3) and (S1) Orexin signaling can speed up hippocampal oscillation, acting as an input signal to the hippocampal clock; (2) The hippocampal oscillator controls the expression of core clock genes; (4) Several key AD-risk genes, (5) including *Bace1/2*, which are regulated through D-boxes and E-boxes, respectively; (6) The rhythmicity of *Bace2* and *ApoE* are altered in the hippocampus of *APP/PS1dE9* mice; (6) and (S2) The orexin precursor gene is rhythmic in the brain. Thus, our model supports the following notions: Orexin signaling influences hippocampal oscillation; the hippocampal oscillator controls the rhythmic expression of AD-risk genes like *Bace1, Bace2*, and *ApoE*, which are the key genes in the metabolism of Aβ; the rhythmicity of these AD-risk genes indicate that disrupted circadian oscillation and sleep both contribute to the risk of AD; and, finally, orexin signaling is involved in reciprocal control of the core clock feedback loop and AD. The blue arrows show the pathways or physiological processes involved in the present study; the black arrows show the well-known pathways or physiological processes; the dashed black arrows represent uncertain relationships between the pathways or physiological processes.

Further expression analysis of the *BACE*s showed that *BACE2* is indeed activated by the *CLOCK:BMAL1* complex. The expression of *BACE1*, which seems to have a substantial phase delay relative to *Bace2* expression, is inhibited by *E4BP4*, another key clock regulator. Additional expression profile data for AD-risk genes in *APP/PS1dE9* mice strengthen our view that AD-risk genes are under the control of the hippocampal circadian clock. We analyzed the expression of *Bace1, Bace2, ApoE*, and *Gsk3α* in the hippocampus and observed that, compared to WT controls, the expression of *Bace2* and *ApoE,* both of which are E-box genes, was elevated in *APP/PS1dE9* mice at ZT5, ZT11, and ZT17. Interestingly, the canonical E-box genes (*Dbp, Per1, Per2*, and *Cry1*) also had altered expression patterns in the hippocampus of *APP/PS1dE9* mice. Old *APP/PS1dE9* mice displayed blunted diurnal variation expression of *Per1* and *Per2* in the hippocampus. These data were consistent with a previous report on the SCN in AD animals^23^. Our results imply that E-box genes are susceptible regulatory mechanisms that relate to the pathology of AD. Therefore, we propose the following network: Orexin signaling influences hippocampal oscillation; simultaneously, the hippocampal oscillator controls the circadian expression of AD-risk genes like *Bace1, Bace2*, and *ApoE,* which are key genes in Aβ metabolism; the rhythmicity of these AD-risk genes indicates that AD is associated with circadian oscillation; finally, orexin signaling is involved in the reciprocal control of the core clock feedback loop and AD (Fig. 7).

In this study, we confirmed that there is an intact hippocampal oscillator; in other words, the hippocampal oscillator contains the molecular components required for SCN-independent, persistent circadian oscillation. We also demonstrated that orexins, which shorten the period of the hippocampal oscillator, are potential inputs to the oscillator. The transcription of AD-risk genes, including *Bace1, Bace2*, and *ApoE*, appear to be outputs of the oscillator. E-box genes such as *Bace2, ApoE, Dbp, Per1*, and *Per2* were found to be more susceptible than genes of other clock subclasses to alterations relating to AD in *APP/PS1dE9* mice. In conclusion, orexin signaling regulates the period of the hippocampal oscillator and circadian oscillation of Alzheimer’s disease-risk genes.

## Additional Information

**How to cite this article**: Ma, Z. *et al*. Orexin signaling regulates both the hippocampal clock and the circadian oscillation of Alzheimer’s disease-risk genes. *Sci. Rep.* **6**, 36035; doi: 10.1038/srep36035 (2016).

**Publisher’s note:** Springer Nature remains neutral with regard to jurisdictional claims in published maps and institutional affiliations.

## Supplementary Material

Supplementary Information

## Figures and Tables

**Table 1:** Cycling parameters of core clock genes predicted by the JTK_CYCLE algorithm.

Gene name	p-value	q-value	period	Phase
*Dbp*	3.854E-05	0.001	24	9
*Bmal1*	3.998E-04	0.005	24	0
*Per1*	0.001	0.010	24	12
*Clock*	0.011	0.052	24	6
*Per2*	0.038	0.108	24	14
*Cry2*	0.038	0.108	24	10

Cutoff: p < 0.05.

**Table 2:** Commonly rhythmic expressed genes in hippocampus under both LD and DD condition.

	hipp_LD and hipp_DD
Common genes	*Gnb5*
	*Sncβ*
	*Casp3*
	Bmal1*
	*ApoE*
	*Cdc2*
	*Bace1*
	Per1*
	*Gng1*
	*Psen2*
	*Gsk3α*
	*Apba1*
	Cry2*
	Per2*
	*Bace2*
	*Prkcδ*

^*^Core clock genes.
