# Supplementary material for: Orexin signaling regulates both the hippocampal clock and the circadian oscillation of Alzheimer’s disease-risk genes
Source: Sci Rep. 2016 Oct 31;6:36035. doi: 10.1038/srep36035 (PMC5086843; doi:10.1038/srep36035)
Supplement: Supplementary Information [file srep36035-s1.pdf]

**Supplementary Information:**

# Orexin signaling regulates both the hippocampal clock and the expression of Alzheimer's disease-risk genes

Zhixiong Ma[1, 2], Weiliang Jiang[3], & Eric Erquan Zhang[2*]

[1]*College of Life Sciences, Beijing Normal University, Beijing 100875, China.*

[2]*National Institute of Biological Sciences, Beijing 102206, China.*

[3]*Department of Gastroenterology, Shanghai First People's Hospital, School of Medicine, Shanghai Jiao Tong University, Shanghai, 200080, China.*

*Corresponding Email: zhangerquan@nibs.ac.cn

## Supplementary Figure Legends:

**Supplementary Figure1: Orexin A shortens the period of hippocampal circadian clock.**

A and B：Orexin A shortens the period of hippocampal circadian clock in a dose dependent manner. (C) pre-orexin A treatment and post-orexin A shows the orexin A shortens the period of hippocampal circadian clock (n=4, * $p < 0.05$, paired T-test). D and E: The EMPA block the effect of orexin B induced shorted period in hippocampus (n=6-10, * $p<0.05$, student's T-test). F and G: The period of hippocampal circadian clock in aging APP/PS1 mice is shorter than the one in the normal WT mice (n=8-18, ** $p< 0.01$, student's T-test)

**Supplementary Figure2: Diurnal expression pattern of *orexins***

(A)Diurnal expression pattern of *orexin precursor* gene in hypothalamus. . Expression orexin precursor mRNA was detected by qPCR in the hypothalamus of WT. the expression of the orexin precursor mRNA is significant different at ZT5 and ZT17 (n=4-5, * $p< 0.05$, student's T-test). (B) Immunostaining of orexin A peptide shows that the diurnal expression pattern of orexin A is consistent with the one of *orexin precursor* gene in later hypothalamus area.

**Supplementary Figure3: The genes are insusceptible to the AD pathology.** (A) Rhythmicity of BACE1 and GSK3α was unchanged in hippocampus of APP/PS1 mice (n=3-5, N.S. $p>0.05$, student's T-test). (B) Rhythmicity of Bmal1, Clock, NR1D1, and NR1D2 was unchanged in hippocampus of APP/PS1 mice (n=4, * $p< 0.05$; N.S. $p>0.05$, student's T-test).

# Figure S1

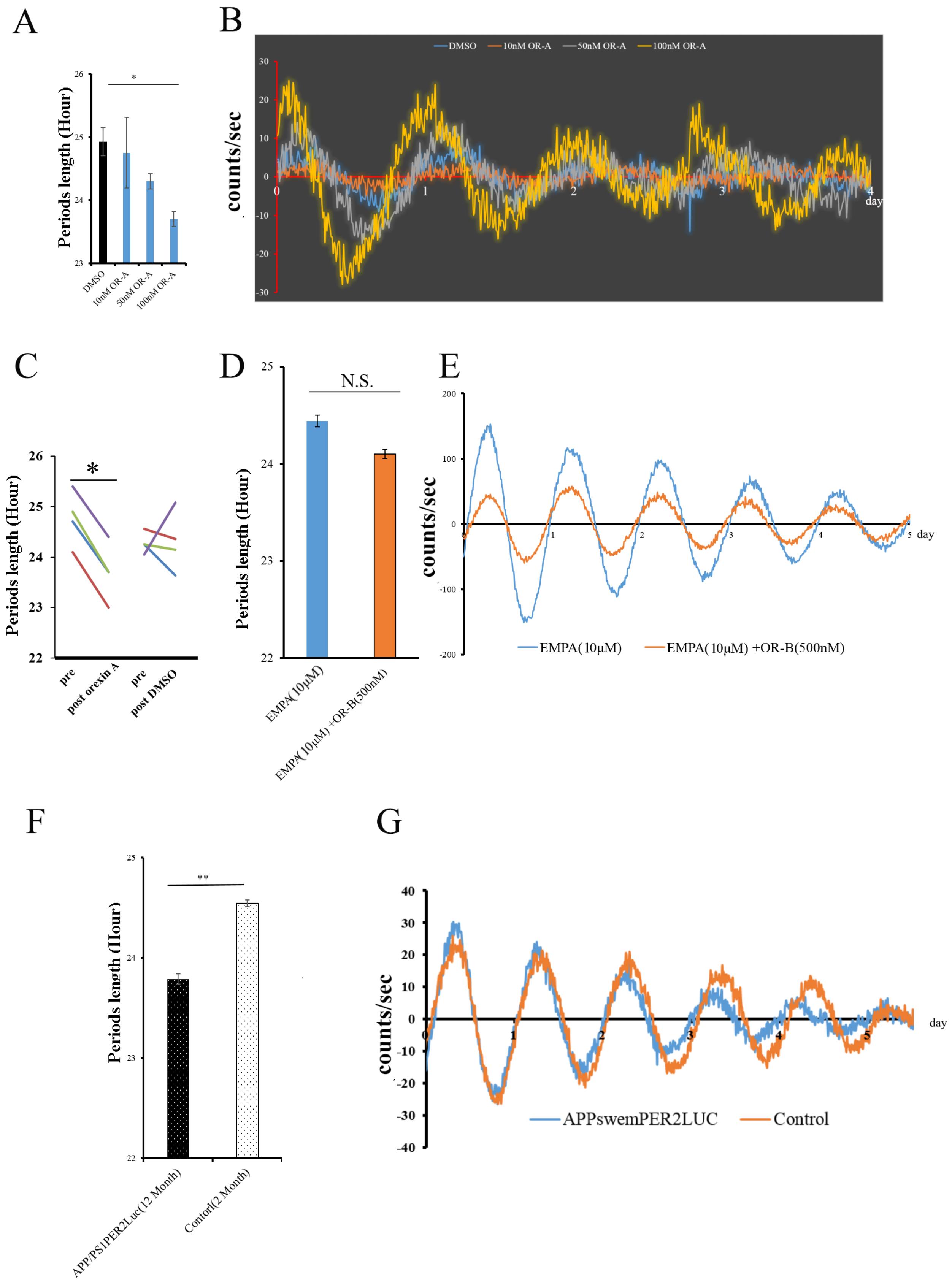
A
B
C
D
E
F
G
Periods length (Hour)
DMSO
10nM OR-A
50nM OR-A
100nM OR-A
counts/sec
day
pre
post orexin A
post DMSO
N.S.
EMPA(10μM)
EMPA(10μM) +OR-B(500nM)
APP/PS1PER2Luc(12 Month)
Contorl(2 Month)
APPswemPER2LUC
Control

# Figure S2

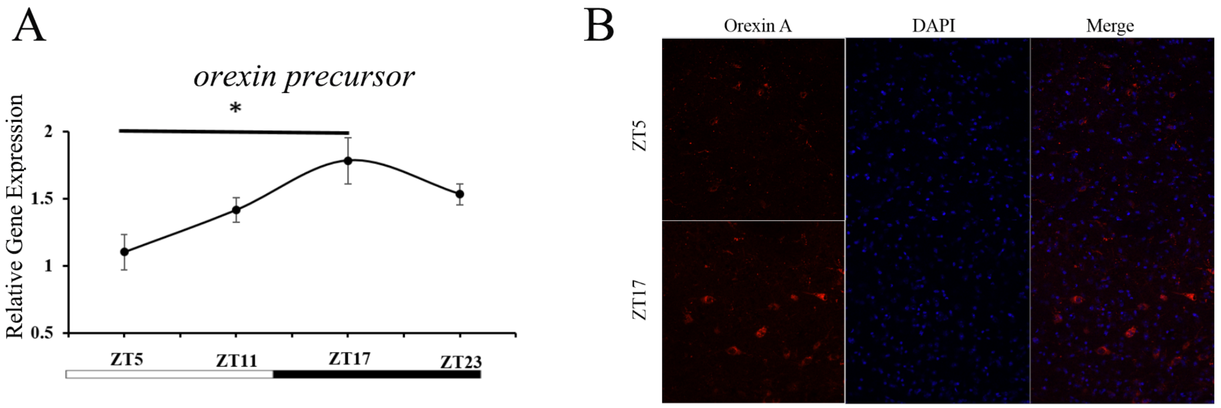

A
orexin precursor
*
Relative Gene Expression
2
1.5
1
0.5
ZT5
ZT11
ZT17
ZT23
B
Orexin A
DAPI
Merge
ZT5
ZT17

# Figure S3

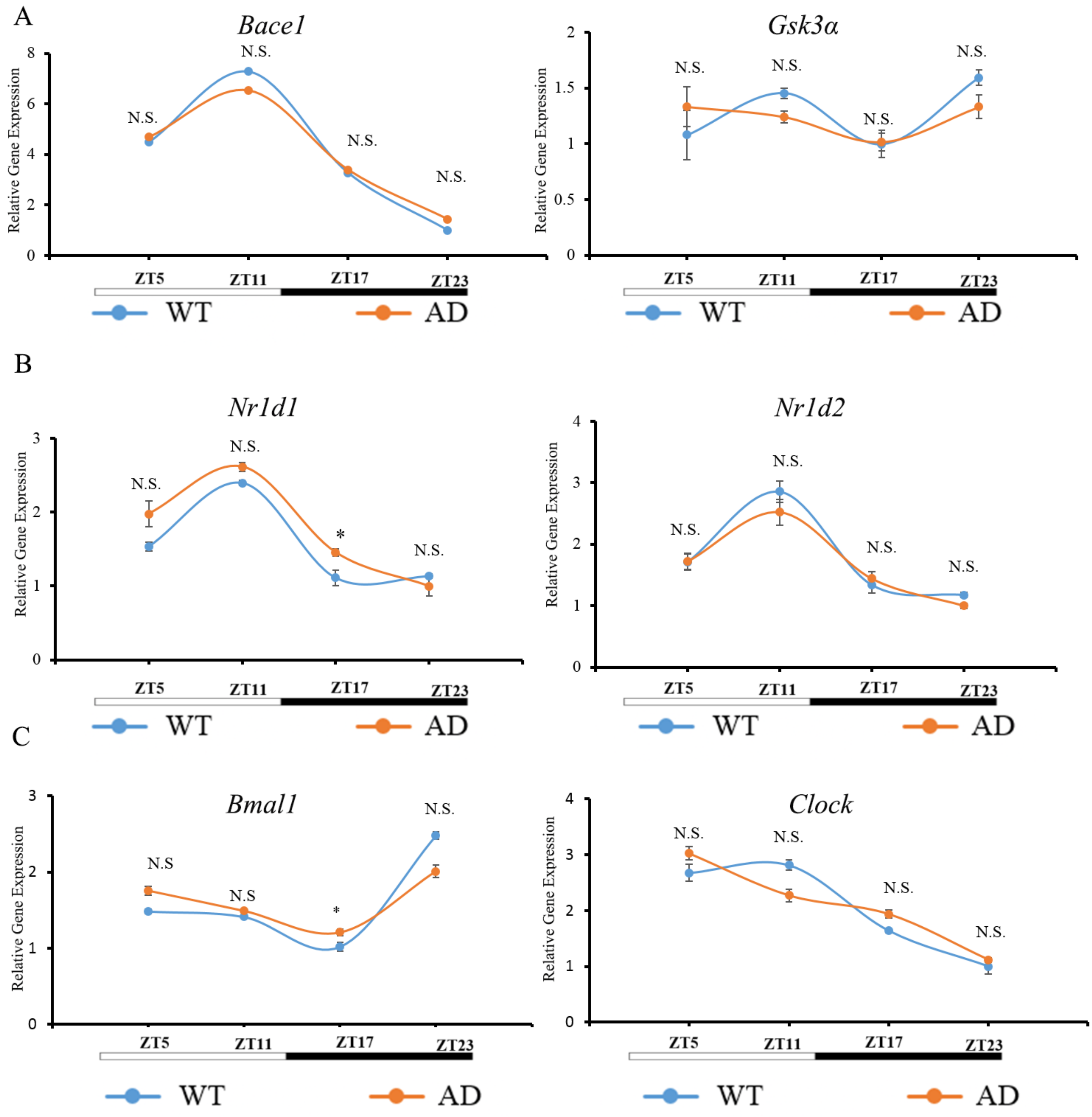

A
Bace1
Gsk3α
B
Nr1d1
Nr1d2
C
Bmal1
Clock
Relative Gene Expression
N.S.
N.S
*
ZT5
ZT11
ZT17
ZT23
WT
AD

**Supplementary Tables:**

**Supplementary Table 1: qPCR primers of AD-risk genes**

| | |
|---|---|
| A2m-1F | TCAAAGTTGCAGTACCAGAG |
| A2m-1R | TCCCATAGGTGTATATGCCA |
| Abca1-1F | GTGTTTCTGGATGAACCAACC |
| Abca1-1R | CTTGACAATGCTTAGGGCAC |
| Ache-1F | CCTGAAGCCCTTAGAGGTG |
| Ache-1R | CTCGTCCAGAGTATCGGTG |
| Adam10-1F | CTGGACATATTTATGGTGAAGAAGG |
| Adam10-1R | CCACGAGTCTTGATGAAACC |
| Adam9-1F | AGATTGCCAGTTCCTTCCA |
| Adam9-1R | GAACCGTTGCAGTACTCAG |
| Apbb1-1F | ATTGTATCCGCCAGCTCTC |
| Apbb1-1R | GCAGATCCTTTCCCTCTCC |
| Apbb2-1F | GGGCAAAGACATGTACCTG |
| Apbb2-1R | GTAAGCAAAGTCCGTCTCAC |
| APH1A-1F | AGCTCCTTAAGAAGGCAGATG |
| APH1A-1R | TGATGATACCGAAGGACAGAC |
| Aplp1-1F | GCGAATGAATCAGAGCCTG |
| Aplp1-1R | AGAAGCTCCTGGATCTGTG |
| Aplp2-1F | GGAAATTGATGAGCTCCTTCAG |
| Aplp2-1R | GGGATCTCCTCACTCTCCT |
| Apoa1-1F | TGTGGATGCGGTCAAAGACA |
| Apoa1-1R | TCCCAGAAGTCCCGAGTCAA |
| Apoe-1F | CACATTGCTGACAGGATGC |
| Apoe-1R | TAATCCCAGAAGCGGTTCAG |
| Apba1-1F | GAATGGAATTATCTGCAGCCT |
| Apba1-1R | ATTTCGATGATCCGATGTCC |
| Apba3-1F | CTCACAGAGACAAGAGAAATCC |
| Apba3-1R | TACTGGTTGTTCCTGTCCC |
| App-1F | ATGTCCCAGGTCATGAGAG |
| App-1R | GATAACGGCCTTCTTGTCAG |
| Bace1-1F | GTTTCCAAGCTCAACATCCTG |
| Bace1-1R | AGTAGCGATGCAGGAAAGG |
| Bace2-1F | CACGAACATCTCTGATTCCA |
| Bace2-1R | GCGTTTCAGAATTTGTCCAG |
| Bche-1F | AAATATGGACATCCCAATGGG |
| Bche-1R | GGGAGCACGAAGTTTAGAG |
| BDNF-1F | TCATACTTCGGTTGCATGAAGG |

| | |
|---|---|
| BDNF-1R | ACACCTGGGTAGGCCAAGTT |
| Capn1-1F | CTATGAGTGGAACAAAGTGGAC |
| Capn1-1R | AACGACATCCAGAACTCCC |
| Casp3-1F | AATGGATTATCCTGAAATGGGC |
| Casp3-1R | GAGCGAGATGACATTCCAG |
| Casp4-1F | CAACAATTGCCACTGTCCA |
| Casp4-1R | TTCTCCAGAGTTCCCACCT |
| CDC2-1F | AAATTGGAGAAGGTACTTACGG |
| CDC2-1R | CTCCTTCTTCCTCGCTTTC |
| Cdk5-1F | GATTGTGAAGTCATTCCTCTTCC |
| Cdk5-1R | TTCAGGTCCCTATGTAGCAC |
| Cdkl1-1F | GCACCAGCAAGTATTTAGC |
| Cdkl1-1R | CAACTCAAGTGTTTCCATGTC |
| Chat-1F | TGGGATCTGGCAACTTCGTC |
| Chat-1R | AGCACCTCCTCACAGCTAGA |
| Clu-1F | ATACCTGCATGAAGTTCTATGC |
| Clu-1R | GGTTTAGAAACTCCTCTAGCTG |
| Ctsb-1F | TATCCCTCTGGAGCATGGA |
| Ctsb-1R | TGGTAAGCAGCCTACATGAG |
| Ctsc-1F | CACCTACCCTGATCTGCTG |
| Ctsc-1R | CTTCTGTTGCTTCCATCACC |
| Ctsd-1F | GATTATCAGAATCCCTCTGCG |
| Ctsd-1R | ATCAGGTCTTCCACAGAGC |
| Ctsg-1F | CTCCAGGAGGTGCAGCTAAG |
| Ctsg-1R | TCCATAGGAGACGATGCCCT |
| CTSL1-1F | GACTGTATGGCACGAATGAG |
| CTSL1-1R | TCTTCTCCCATATCGCTCTC |
| Ep300-1F | TGCGTCTGTAGAGCTGTGAG |
| Ep300-1R | TCTCTTCTCCGAAACGGGGT |
| Ern1-1F | AAGAAGATCCAGTCCTGCA |
| Ern1-1R | AAAGGGAAGTTTCGTCAGG |
| Gap43-1F | GGAGAAAGACGCTGTAGAC |
| Gap43-1R | CATGTTCTTGGTCAGCCTC |
| Gnao1-1F | CACTTCAGGCTGTTTGACG |
| Gnao1-1R | TGGAGTCGAAGAGCATGAG |
| GNAZ-1F | GGCAGAGGTCAGAACGCAAA |
| GNAZ-1R | GCTCGTCTGGTTGTCCTCATA |
| Gnb1-1F | CCACAAACAAGGTTCATGC |
| Gnb1-1R | CCACATAATTCCCAGAAGGAG |

| | |
|---|---|
| Gnb2-1F | ACACTGACCCAGATCACAG |
| Gnb2-1R | ACCTAGACATACTAAAGCAGGG |
| Gnb4-1F | GGGCTCCAGCTCTTCACTTG |
| Gnb4-1R | GAGGGATGGCGTGCATCTTA |
| Gnb5-1F | GTCTCCATCCTGTTTGGAC |
| Gnb5-1R | ATGCCCAAACTCTTAGGGT |
| Gng11-1F | CTTCACATCGAGGATCTGC |
| Gng11-1R | TTTAGATACCTGTTGTCTCTGC |
| Gng3-1F | TATGAGTATTGGTCAAGCACG |
| Gng3-1R | GCCTTGGACACCTTTATCC |
| Gng4-1F | GACCCTGAGTCAGCTTCTC |
| Gng4-1R | TTCATTCCTGCACTCCCTG |
| Gngt1-1F | AAAAACAGCTTCCCTGACAGAA |
| Gngt1-1R | AGCCTCCTTTGAGTTCCTTGA |
| Gngt2-1F | TGAGAAGGAGCTGTTGAGG |
| Gngt2-1R | CCTGTCTTGGAAATCAGATCAC |
| GSK3A-1F | AAGCTCTGCGATTTTGGCAGT |
| GSK3A-1R | GAGTTCTGGAGCACGGTAGTA |
| Gsk3b-1F | CTTGGACAAAGGTCTTCCG |
| Gsk3b-1R | AATGTGCACAAGCTTCCAG |
| Hsd17b10-1F | GTGGCCATTAAGACATACCAC |
| Hsd17b10-1R | AAGATTCACATTGATAACCCGC |
| Ide-1F | TGCATCAGGGATGAATGCA |
| Ide-1R | AAGAGATCGCATATAGGCCTC |
| Il1a-1F | ATGATCTGGAAGAGACCATCC |
| Il1a-1R | CGAGCTTCATCAGTTTGTATCTC |
| Insr-1F | TCAGAAAGTTTGCCCAACC |
| Insr-1R | GTCATCAGGTTCCGAACAG |
| Lpl-1F | AGAATTACTGGTTTGGATCCAG |
| Lpl-1R | AAATCAGCGTCATCAGGAG |
| Lrp1-1F | TGTATGAAGGTGGAGAGCC |
| Lrp1-1R | AGTTGGTAGGCTTGTCAGG |
| Lrp6-1F | GAGGAAGATCTTGATTTCAGAGG |
| Lrp6-1R | GTCCAATACATGTACCCAACC |
| Ntrk1-1F | CATACACAGACGCTTCATGAC |
| Ntrk1-1R | TGTGGGTACACCACAACTG |
| Ntrk2-1F | AGAACGAGTATGGGAAGGA |
| Ntrk2-1R | TTGGGTTTGTCTCGTAGTC |
| Pkp4-1F | TCCATGAAAGTGAGGGATCG |

| | |
|---|---|
| Pkp4-1R | CTTCCTGGTACCCACTGTC |
| Plat-1F | TTATTGTCGGAATCCAGATGGT |
| Plat-1R | TCACAGTATTCCCACGTCAG |
| Plau-1F | ACAATTACTGCAGGAACCC |
| Plau-1R | ATTCTTGGACAAACTGCCT |
| Plg-1F | CCACGTACCCAACTACTCTC |
| Plg-1R | TCATCATTGTCTGGGTTCCT |
| Prkca-1F | AGTCGGGAAATTTAAGGAGC |
| Prkca-1R | TTCAGATCCCTGTAAATGATCC |
| Prkcb-1F | AGAACCACAAATTCACCGC |
| Prkcb-1R | AAAGCAGCAGACTTGACAC |
| Prkcd-1F | TGAGTTCTGGCTGGATCTG |
| Prkcd-1R | GTTTAATGGCTCCACGACG |
| Prkce-1F | TTTGGCAAGGTCATGTTGG |
| Prkce-1R | CGTCTTGTAGGATAACGTCCT |
| PRKCG-1F | CCTGCAATGTCAAGTCTGTAG |
| PRKCG-1R | ACACTCGAAGGTCACAAATTC |
| Prkci-1F | ACGATGAGGATATCGATTGGG |
| Prkci-1R | CTGGAAGCAAGAATGCAGC |
| Prkcq-1F | AACCGTGGAACTCTACTCC |
| Prkcq-1R | TGAGGTTTCAGCTCTAACCA |
| Prkcz-1F | GTGCAGCGAAAGGATATGG |
| Prkcz-1R | GACAGAATCCATATGCCTCCT |
| Psen1-1F | GCTCTTATCTATTCCTCAACAATGG |
| Psen1-1R | CTTGGGTACCCTCCTTTGG |
| Psen2-1F | CAGAGATGGAAGAAGACTCCT |
| Psen2-1R | AAGATGAAGTCTCCCAGGC |
| Snca-1F | GAGTCCTCTATGTAGGTTCCA |
| Snca-1R | CTTGCTCTTTGGTCTTCTCAG |
| Sncb-1F | CTATGTCGGAAGCAAGACCA |
| Sncb-1R | CAGAGAACACAGCTCCTCC |
| Ubqln1-1F | AATCCTCAGCTGCAGAGTC |
| Ubqln1-1R | TTCACGGTTCAAGAATCCCA |
| Uqcrc1-1F | CTTTCAAGGGAACAAAGAATCG |
| Uqcrc1-1R | TGTAGGCATTAAGATGAGCC |
| Uqcrc2-1F | AGCAGGCAGTAGATATGAGG |
| Uqcrc2-1R | CCTTTGGTAGTCAAACTAGATGC |
| mINS-1 F1 | CACCCCACCTGGAGACCTTA |
| mINS-1 R1 | CCACACACCAGGTAGAGAGC |

**Supplementary Table 2: qPCR primers of core clock genes**

| | |
|---|---|
| mDBP_mrna-f | CCTGAGGAACAGAAGGATGA |
| mDBP_mrna-r | ATCTGGTTCTCCTTGAGTCTTCTTG |
| mCRY2-F1 | GCCTCTGCCTCCCTTGATTT |
| mCRY2-R1 | TGACATAAGCAGCATGGGCA |
| mCLOCK-F1 | CACGCGCTCCCGTGAAAG |
| mCLOCK-R1 | GTCCTTGTCATCTTCTTCCACCA |
| mCRY1-F2 | AGCTCGTGTCCGTTCGTG |
| mCRY1-R2 | CCTGCGCTTCCAAGAAAACC |
| mBMAL1_mrna-f | CTCGACACGCAATAGATGGGA |
| mBMAL1_mrna-r | CTTCCTTGGTCCACGGGTT |
| mPER1_mrna-f | TGAAGCAAGACCGGGAGAG |
| mPER1_mrna-r | CACACACGCCATCACATCAA |
| mPER2_mrna-f | GAAAGCTGTCACCACCATAGAA |
| mPER2_mrna-r | AACTCGCACTTCCTTTTCAGG |
| mNR1D1 qPCR F | TGGCATGGTGCTACTGTGTAAGG |
| mNR1D1 qPCR R | ATATTCTGTTGGATGCTCCGGCG |
| mNR1D2 qPCR F | GGAGTTCATGCTTGTGAAGGCTGT |
| mNR1D2 qPCR R | CAGACACTTCTTAAAGCGGCACTG |

**Supplementary Table 3: qPCR primers of housekeeping genes, *orexin precursor* gene and orexin receptors**

| | |
|---|---|
| mActin_mrna-nf | GGCTGTATTCCCCTCCATCG |
| mActin_mrna-nr | CAGTTGGTAACAATGCCATGT |
| mGapdh_mrna-nf | GACCTCAACTACATGGTCTACA |
| mGapdh_mrna-nr | ACTCCACGACATACTCAGCAC |
| mOrexin-PreF1 | CCTGCCGTCTCTACGAACTG |
| mOrexinPreR1 | GGTGCTAAAGCGGTGGTAGT |
| Hcrtr1 F3 | CTGTGGCGCGATTATCTCTAC |
| Hcrtr1 R3 | GCCAGGGACAGGTTGACAA |
| Hcrtr2 F3 | GAGGATTCCCTCTCTCGTCG |
| Hcrtr2 R3 | GGTGTAGGTATTCCCTCCACA |

**Supplementary Table 4: Primers for amplified the coding sequence of E4BP4 and the promotor regions of *BACE1* and *BACE2***

| | |
|---|---|
| Human E4BP4 F | ACCGGCGGCCGCATGCAGCTGAGAAAAATGCA |
| Human E4BP4 R | ACCGGGCGCGCCTTACCCAGAGTCTGAAGCAG |
| BACE1 pro gen-REV R | AGCTAAGCTTTTGGGCCAAGGTGGGCCCCG |
| BACE1 pro 1k | ATCCGGTACCTTGCAGCCTGGAAAAACTCT |
| BACE2 pro gen-REV R | AGCTAAGCTTGTGCGCCCAAGCCCACGGCG |
| BACE2 pro 1.4k | ATCCGGTACCTTGTTTTTTAAAACAGCCAACATAA |
